# The flora of south-eastern Mato Grosso State (Brazil): a review of herbarium collections

**DOI:** 10.3897/BDJ.9.e72907

**Published:** 2021-11-10

**Authors:** Erica Pereira Campos, Massimo Giuseppe Bovini, Ariane Luna Peixoto

**Affiliations:** 1 Universidade Federal de Rondonópolis, Rondonópolis, Brazil Universidade Federal de Rondonópolis Rondonópolis Brazil; 2 Instituto de Pesquisas Jardim Botânico do Rio de Janeiro, Rio de Janeiro, Brazil Instituto de Pesquisas Jardim Botânico do Rio de Janeiro Rio de Janeiro Brazil

**Keywords:** biodiversity database, Cerrado, collection effort, endangered species, flora of Brazil

## Abstract

The diversity and richness of the Brazilian flora are documented in herbarium collections. When areas are deforested, but not documented, we lose both flora and the opportunity to know which species occupied those areas. The south-eastern mesoregion of Mato Grosso State, comprising 22 municipalities, has undergone the loss of native vegetation cover, accelerated by the substantial expansion of agribusiness. For an in-depth look at the consequences and the current state of the flora in that mesoregion, we consulted both speciesLink and JABOT databases. More specifically, we carried out a comprehensive study of herbarium collections by municipality and compiled data including scientific name, collection date, collection locality, collector name and observations made during collection. We obtained 5,861 records of angiosperms, ferns and lycophytes of 160 families, 770 genera and 1,792 species. The specimens are deposited in 80 herbaria of which the Herbarium of Universidade Federal de Mato Grosso (UFMT) hosts the largest collection. Rondonópolis was the most sampled municipality, whereas 50% of the municipalities were poorly sampled and one showed no collection records at all. Amongst the species occurring only in Mato Grosso, eleven were collected only in that mesoregion and twenty species were under some degree of threat. Our study recommends increasing collection and identification efforts to enable effective conservation actions and improve richness estimates for the State of Mato Grosso and the Cerrado, in general, before more of the flora is lost to deforestation.

## Introduction

Brazil is recognised as one of the richest countries in plant diversity. The Brazilian Flora has recorded the occurrence of approximately 38,676 species of vascular plants and bryophytes of which Angiosperms correspond to over 90% of the total (Flora do Brasil 2020). Available data also show that approximately 57.4% of Angiosperm species are endemic to the country (BGF - The Brazil Flora Group 2015).

Specimens records are documents that certify flora diversity and richness of a given region or country and are deposited in botanical collections. These collections exist of living or herbarium material and their associated data (Peixoto and Morim 2003).

According to Willis et al. (2003), botanical collection data are often the only available data for a given species and qualify as a fundamental tool for assessing the conservation status of a species. The analysis of the information contained in the exsiccatae labels, encompassing geographical and ecological aspects, can support several studies. Any biodiversity or systematic study largely depends on the quality and representation of herbarium collections (Shepherd 2003). One of the main purposes of herbarium collections is to serve as taxonomic repositories: storing specimens allows users to return to them over decades and centuries to check the identification of plants and study the characteristics of the given species, reference collections used to identify species and describe new species (Greve et al. 2016).

Besides documenting the country’s biological diversity, the specimens deposited in herbaria keep part of the history of regions previously covered by natural vegetation, which are now occupied by cities, various enterprises or which are now deforested (Peixoto et al. 2009).

Without the existence of voucher materials in herbaria, it is impossible to verify whether observations are correctly identified, to assess the geographic distribution, carry out species distribution modelling analysis, study the phylogeny or even define the conservation status of a species (Windisch and Peralta 2010) and priority areas for conservation using floristic criteria. Furthermore, botanical knowledge is essential for vegetation, ecology and conservation studies (Pott and Pott 1999).

According to MacDougall et al. (1998), herbaria data provide two types of information especially relevant to conservation planning: location of species occurrence, which indicates where they were found and may persist and habitat descriptions for each species. To preserve such information, the herbarium is a valuable resource, capable of storing comprehensive data of individual plants and representatives of species, as well as natural or cultivated populations. Thus, at its foundation, the most obvious function performed by herbaria is that of documenting botanical studies, especially taxonomic and floristic ones (Monteiro and Siani 2009).

In Brazil, 216 active herbaria are distributed in all states of the Federation. In the Midwest region, there are 21 herbaria and for the State of Mato Grosso, six active herbaria (Gasper et al. 2020, Rede Brasileira de Herbários 2020).

The south-eastern mesoregion of Mato Grosso is composed of 22 municipalities (Miranda and Bastos 2016). It is an area with a strong presence of farming and cattle breeding (Borges et al. 2014). Areas with remnant natural vegetation are fragmented, generally located in difficult to access sites and rugged relief that are hard to transform into pastures or crops.

Owing to the rapid loss of native vegetation cover and the scarcity of studies (SEMA - Secretaria de Estado de Meio Ambiente 2021), it is essential to assess the current state of knowledge of the region’s vegetation to support and guide actions for the recovery and conservation of the local flora.

Thus, the following questions guided our study:

1. How are family, genus and species diversity represented in the south-eastern mesoregion of the State of Mato Grosso?
2. What is the frequency of occurrence records?
3. Where are the collections spatially concentrated?
4. Does the data collection of specimens sampled in south-eastern Mato Grosso enable defining the poorly-sampled areas that still hinder the study of local flora?
5. Are any endangered or extinct species found in the region?

## Material and methods

### Study area

Mato Grosso consists of 903,329.7 km^2^ and occupies 56.23% of the Brazilian Midwest region, bordering the States of Amazonas, Pará, Tocantins, Goiás and Mato Grosso do Sul and sharing its borders with Bolivia (Miranda and Bastos 2016). Its climate is megathermal, tropical continental, alternately humid (spring-summer) and dry (autumn-winter) (Tarifa 2012).

Vegetation consists of elements from the Amazon phytogeographic region, which occupies approximately 53.5% of Mato Grosso’s territory, followed by the Cerrado and Pantanal biomes with 39.7% and 6.8%, respectively (Borges et al. 2014).

**Figure 1.**
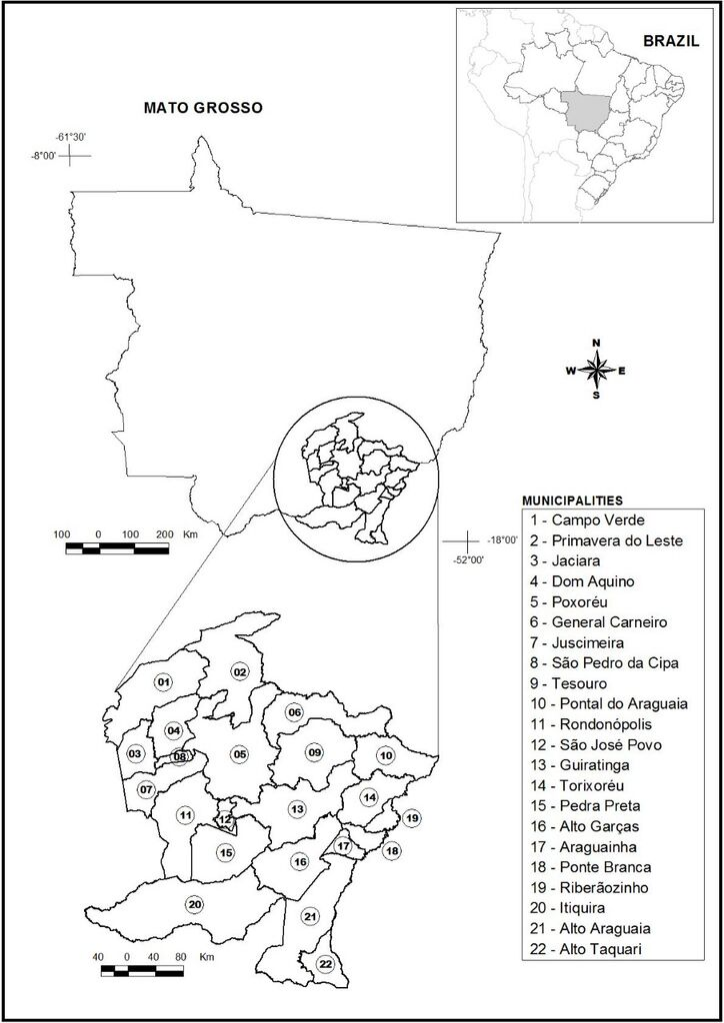
Geographic location of the municipalities of the south-eastern mesoregion of Mato Grosso.

The State is currently divided into five geographic mesoregions: north, north-eastern, south-western, mid-southern and south-eastern (Miranda and Bastos 2016). The south-eastern mesoregion of Mato Grosso occupies an area of 71,847.7 km^2^ and comprises 22 municipalities within the Cerrado domain: Alto Araguaia, Alto Graças, Alto Taquari, Araguainha, Campo Verde, Dom Aquino, General Carneiro, Guiratinga, Itiquira, Jaciara, Juscimeira, Pontal do Araguaia, Ponte Branca, Poxoréo, Primavera do Leste, Ribeirãozinho, Pedra Preta, Rondonópolis, São Jóse do Povo, São Pedro da Cipa, Tesouro and Torixoréu (Miranda and Bastos 2016) (Fig. 1).

The soils of the south-eastern mesoregion vary between Dark-Red and Red-Yellow Latosols, Red-Yellow Podzolic, Cambisols and Quartz Sands (SEPLAN - Secretaria de Estado e Planejamento e Gestão 2019).

### Data collection

The databases from *species*Link (http://inct.splink.org.br) and JABOT (Botanical collections management system of the Rio de Janeiro Botanical Garden - JBRJ) (http://jabot.jbrj.gov.br) networks, with records up to December 2019, were consulted. Data from the Global Biodiversity Information Facility (GBIF) (GBIF Secretariat 2019) network for herbaria abroad that were integrated into the *species*Link network were also used.

For each of the 22 municipalities studied, we surveyed the herbaria containing records and compiled information from these records, including family, scientific name, collection date, collection locality, collector name, geographic coordinates and observations made during collection. Indigenous lands located within these municipalities were also assessed.

We created a spreadheet in EXCEL (version 2016) to facilitate information organisation, checking and editing duplicate records; 186 records were discarded, which included only the name of the state, but no information about the municipality or location that could support the inclusion of the sample in one of the municipalities in the south-eastern mesoregion of Mato Grosso. We also excluded 269 records containing no date and followed the same procedure for samples without specific determination. We used ESRI ARCGIS, v.10.5 and ARCVIEW GIS 3.2 software to prepare maps.

Herbaria with fifty or more records of the studied area were considered significant (Table 1). We considered 200 collections or more as significant for the municipality.

Families were listed following APG IV (APG 2016). Scientific names were checked and updated following the Flora do Brasil 2020 (http://floradobrasil.jbrj.gov.br) and, whenever necessary, specialists for the specific group were consulted, as in the case of *Chaetothylaxtocantinus* Nees which was changed by *Justiciatocantina* (Nees) V.A.W. according to the specialist in Acanthaceae, Dr. James Lucas da Costa Lima.

The presence of endangered species was verified by accessing the Brazilian National Center for Flora Conservation website (CNC Flora) (www.cncflora.jbrj.gov.br/portal), a national reference for the conservation of endangered Brazilian flora.

## Results and discussion

### Taxonomic diversity

The inventory showed a total of 5,861 records of angiosperms, ferns and lycophytes distributed amongst 160 families, 770 genera and 1,792 species and infraspecific categories (Suppl. material 1). Of the total number of records, 5.59% were identified up to the family and 17.64% up to the genus, the remaining specimens being identified up to species level.

**Figure 2.**
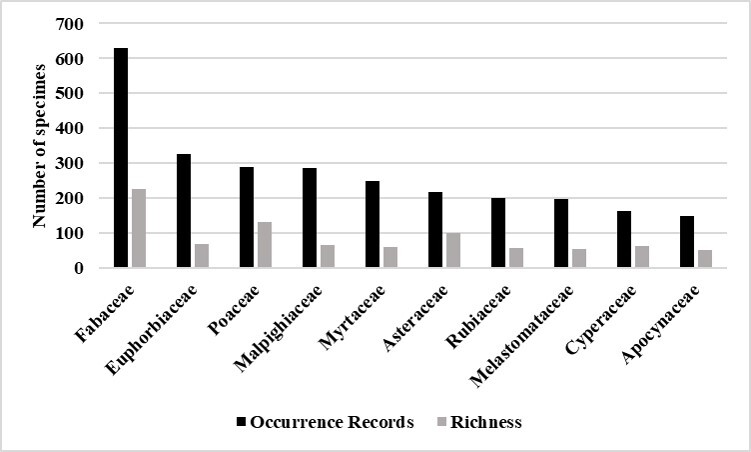
The ten most collected families and their respective species richness in the south-eastern mesoregion of Mato Grosso.

The ten families with the highest number of occurrence records and the highest richness were those already recorded in other studies in Cerrado areas (Ratter 1997, Mendonça et al. 2008,Campos et al. 2018). Fabaceae remained the most collected family and showed the highest species richness. Euphorbiaceae was the second most recorded family and the fourth in number of species, while Poaceae was the second in richness, despite being the third most collected family (Fig. 2). The percentage of families represented by a single species was 26.9% or 43 families.

The data confirmed that these ten families are amongst the largest families in the Brazilian flora in number of species, as well as in the Cerrado (BGF - The Brazil Flora Group 2015), contributing 47.2% to the floristic richness of the region.

The most abundant genera are *Paspalum* (27), *Eugenia* (23), *Myrcia* (23), *Byrsonima* (21) and *Rhynchospora* (18). Except for *Byrsonima* (BGF - The Brazil Flora Group 2015), these genera are amongst the 30 largest angiosperm genera in Brazil.

The most collected angiosperm species were *Heveabrasiliensis* (Willd. ex A. Juss.) Müll. Arg (64 occurence records observed), *Duguetiafurfuracea* (A. St.-Hil.) Saff. (38), *Byrsonimaintermedia* A. Juss. (24), *Kielmeyeracoriacea* Mart. & Zucc. (21), *Palicoureacoriacea* (Cham.) K. Schum. (21), *Myrciaguianensis* (Aubl.) DC. (20), *Rauvolfiaweddeliana* Müll. Arg. (19), *Davillaelliptica* A. St.-Hil. (18), *Xylopiaaromatica* (Lam.) Mart. (17), *Eugeniaaurata* O. Berg (16), *Manihotcezarii* M. Martins (16) and *Miconiaalbicans* (Sw.) Steud. (16). Ferns were represented by *Pityrogrammacalomelanos* (L.) Link (20) and lycophytes by *Palhinhaeacamporum* (B. Øllg. & P.G. Windisch) Holub (17). Our data also showed that 57.8% of species were collected only a single time, revealing the need for a significant increase in fieldwork to collect and identify the material deposited in collections.

It is worth highlighting that the species with the highest number of collections, *H.brasiliensis* (rubber tree), is mainly native to the states of northern Brazil with no natural occurrence in Mato Grosso (Flora do Brasil 2020). It is considered a cultivated species in the region and, in the present study, all specimens came from a rubber tree plantation in the District of Ouro Branco, Municipality of Itiquira. The other species mentioned are widely distributed in the Cerrado in several states, except for *Rauvolfiaweddeliana* (Apocynaceae), the occurrence of which is confirmed only in the States of Mato Grosso and Mato Grosso do Sul (Flora do Brasil 2020).

### Herbarium collections

**Figure 3.**
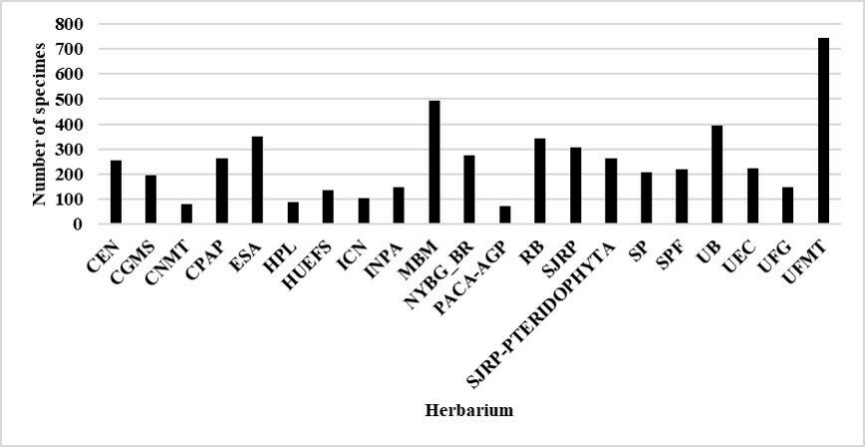
Herbaria with fifty or more records of specimens from the south-eastern mesoregion of Mato Grosso.

The records of flora species from the south-eastern mesoregion of Mato Grosso are deposited in eighty herbaria in Brazil and abroad. The Herbarium of the Federal University of Mato Grosso (UFMT) contains the largest collection (744 records), both in number of records and species diversity of the studied area. The second-largest collection is housed in the Municipal Botanical Museum of Curitiba (MBM) (495 records) and the third is in the Herbarium of the University of Brasília (UB) (395 records) (Fig. 3).

The botanist Gerdt G. Hatschbach, founder of the MBM, was the primary collector in Brazil, as well as amongst the most prominent in the number of collections. His records in the region cover the period between 1966 and 1998.

### Collection periodicity

The oldest collection record in the region is a fern species with wide distribution in the country, *Didymoglossumpunctatum* (Poir.) Desv. (Hymenophyllaceae), collected in the Alto Araguaia region by Carl August Wilhelm Schwacke in 1877 and deposited in the Jardim Botânico do Rio de Janeiro Herbarium (RB - barcode 00650249) (Forzza et al. 2021). In the 1910s, records were only found for the year 1911 and those were represented by the collections of Frederico Carlos Hoehne, comprising one specimen of family Fabaceae (*Calopogoniumcaeruleum* (Benth.) C.Wright) and five Orchidaceae specimens (*Cyrtopodiumpaludicola* Hoehne), all deposited in the R herbarium (Museu Nacional, Rio de Janeiro, Brazil) (Silva et al. 2021) and collected in Itiquira. None of these records showed any additional information about collection location.

All records between 1920 and 1929 occurred in 1926, comprising 19 Poaceae and one Apocynaceae species. All collections were made by Marshal Cândido Mariano da Silva Rondon (1865-1958).

Between 1900 and 1930, the Rondon Commission was created, responsible for constructing telegraph lines all over Brazil (Sá et al. 2008). During this period, the Commission for the Construction of Strategic Telegraph Lines from Mato Grosso to Amazonas (CLTEMTA) was created and work continued between 1907 and 1915. One of the objectives of CLTEMTA was the scientific exploration of the territory. Therefore, scholars and naturalists of diverse areas, such as cartography, geology, anthropology, zoology and botany, joined the Commission. Amongst the botanists, Frederico Carlos Hoehne and João Geraldo Kuhlmann stand out, as both worked for the Commission between 1908 and 1915 (Sá et al. 2008). The aforementioned botanical collections made by Marshal Rondon seem to evidence his proximity to natural history.

The Rondon Commission played an important role in the natural history of the State of Mato Grosso, which, at the time, occupied much more territory. According to Sá et al. (2008), the study of the botanical material collected during the project included the participation of several foreign specialists. At the time, were no specialists for most taxonomic groups collected in Brazil. Moreover, specialised literature and type material for comparison in Brazilian herbaria were largely absent. Hoehne, who was responsible for organizing the botanical specimens, was concerned about disseminating the results of such an important project and decided to send part of the material abroad to be studied by specialists (Sá et al. 2008). Unfortunately, the route of the Rondon Commission did not include the south-eastern region of the State of Mato Grosso which could explain the small number of records between 1900-1930.

**Figure 4.**
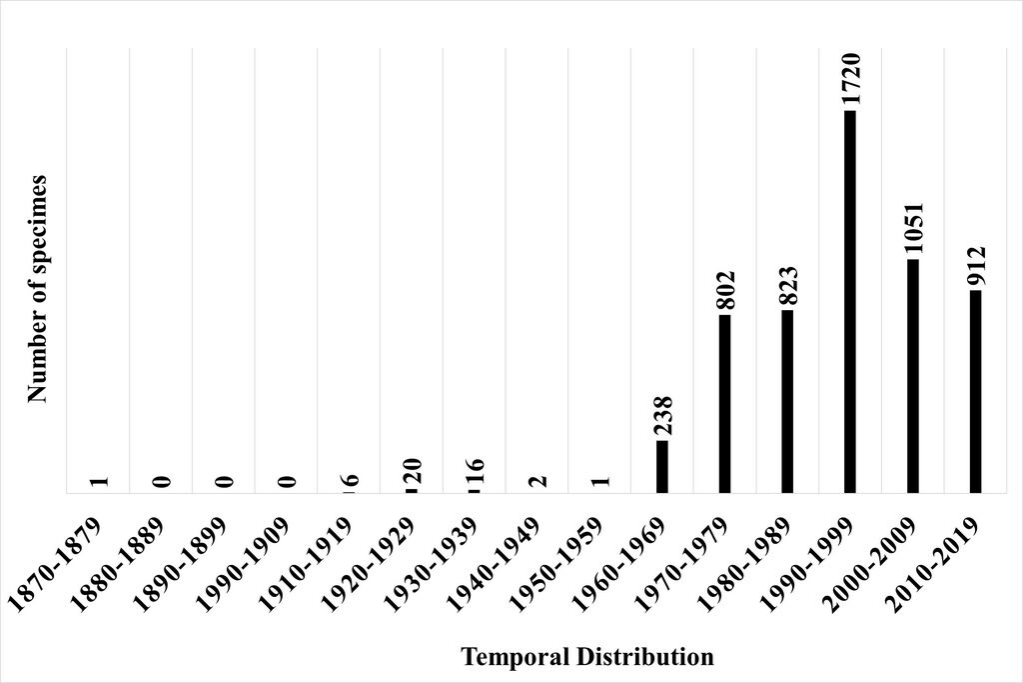
Temporal distribution of botanical collections in the south-eastern mesoregion of Mato Grosso.

The following decades saw a significant recovery in collections, which started in the 1960s, but peaked between 1990 and 1999. With the consolidation of undergraduate and graduate courses in several institutions, it is likely that academics from UFMT and researchers from Empresa Brasileira de Pesquisa Agropecuária (EMBRAPA- Pantanal) went into the field to carry out their studies, resulting in an increase in records deposited in herbarium collections (Fig. 4).

### Spatial characterisation of collections

The Municipality of São Pedro da Cipa was the only one with no collection. Rondonópolis, the largest municipality in the region, was sampled the most (1,116 collection records), followed by Alto Araguaia (956), which borders the state of Goiás and Itiquira (502), which borders the State of Mato Grosso do Sul. The three municipalities have a good highway network, providing easy access.

**Figure 5.**
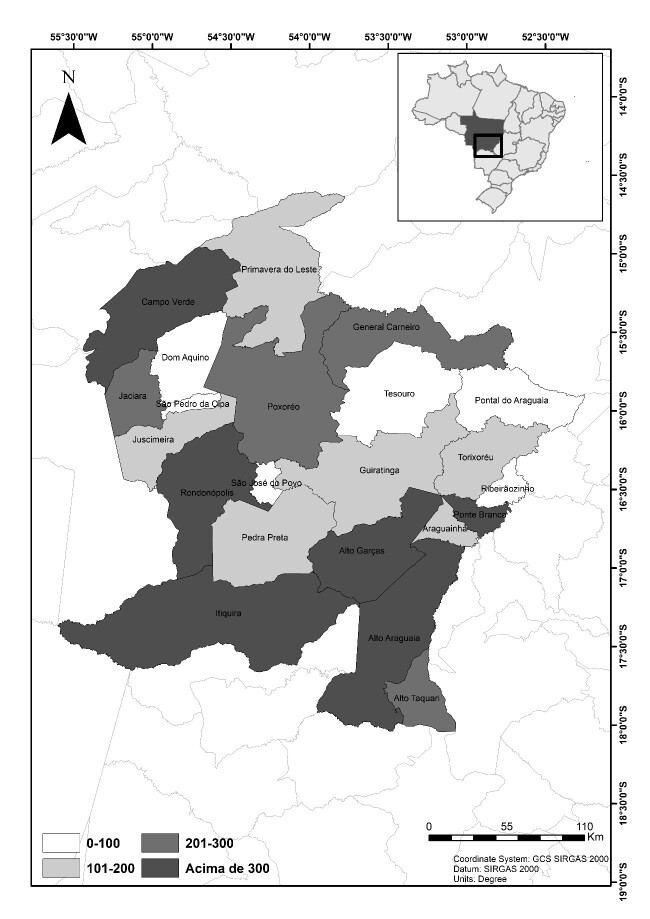
Municipalities of the south-eastern mesoregion of Mato Grosso represented in shades of grey according to the number of records.

Considering a minimum number of 200 records per municipality, it was possible to evaluate a rather high percentage of municipalities (50%) having a poorly-collected area, i.e. less than 200 samples deposited in collections (Fig. 5).

**Figure 6.**
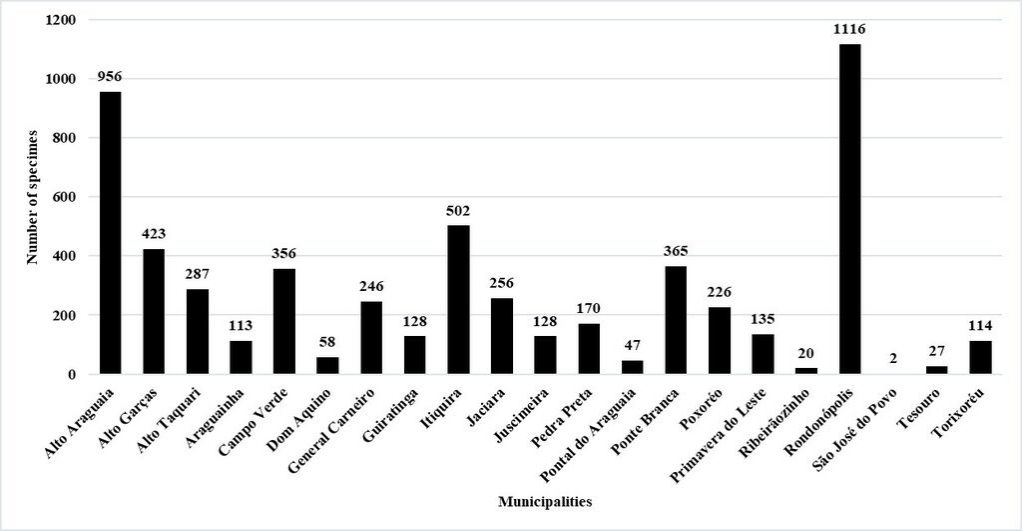
Number of occurrence records of collections per municipality in the south-eastern mesoregion of Mato Grosso.

The Rondonópolis region is strategically located at the junction of highways BR-163 and BR-364, connecting the northern and southern regions of Brazil. Thus, the easy access, good infrastructure and the Biological Sciences course of the Federal University of Rondonópolis may have contributed to the higher number of records (Fig. 6).

Most collections in the south-eastern mesoregion of Mato Grosso occurred along roads, followed by those along watercourses. Protected areas represent 1.47% of the total records with 13 species collected exclusively in these areas (Table 2). The present study showed poor floristic knowledge, reinforcing the need to intensify floristic sampling in these areas.

According to Barbosa and Vieira (2005), the northern and Midwest regions of Brazil have the lowest number of herbaria and the smallest set of specimens in collections, not reaching one sample/km^2^. Therefore, these regions face major challenges. Although they represent an important part of the Brazilian territory, concentrating different natural ecosystems, they have the lowest collection rates and the lowest number of specialists and herbaria.

Gasper et al. (2020), using 206 datasets of active herbaria on the INCT Virtual Herbarium of Flora and Fungi platform, reported that the records per km^2^ are much lower in the North (0.2), Midwest (0.45) and Northeast (0.88) when compared to the Southeast (1.85) and South (2.01) regions.

However, the number of records found in the present study (of 0.081 specimens/km^2^) is lower than those cited by either Barbosa and Vieira (2005) or Gasper et al. (2020) for the Midwest region. However, it is above the estimate made by Shepherd (2003) (0.015 collections/km^2^) for the State of Mato Grosso. This author recommended three collections/km^2^ as sufficient sampling for an adequate effort to know the flora of a region.

### Endemic and endangered species

Of the 166 species mentioned in Flora do Brasil (2020), with confirmed occurrence only in Mato Grosso, 6.6% (11) were collected in the south-eastern region of the State and of these, three were collected exclusively in this region: *Blepharodonhatschbachii* Fontella & Marquete (Apocynaceae), *Mikaniasarcodes* Baker (Asteraceae) and *Onoserisbrasiliensis* Cabrera (Asteraceae). These results indicate low effort in collecting and identifying botanical material to check for the presence of other possible endemic species.

In addition, we found twenty species under some degree of threat (Table 3), twelve of which were collected only once. Five of these species were considered by Red List Authority for plants in Brazil - CNC Flora endangered (EN), eight vulnerable (VU) and seven near threatened (NT)

The present study indicates *Blepharodonhatschbachii* Fontella & Marquete (Apocynaceae) as a priority species for conservation. Besides being endangered (EN), it has confirmed occurrence only in Mato Grosso with collections in two municipalities of the south-eastern mesoregion in the years of 1973 and 1974 (GBIF Secretariat 2019,Reflora - Herbário Virtual 2020).

## Conclusions

The south-eastern mesoregion of Mato Grosso is composed of 22 municipalities and shows a remarkable species richness and diversity, as documented in herbarium collections. Therefore, we want to stress the importance of inventorying and characterising local flora species and their populations. The consulted herbarium collections contained 1,792 species of this region, with many undetermined collections. Unfortunately in this study, a large number of municipalities showed scarce records and locality information.

Therefore, we want to stress the importance of increasing the efforts to collect and identify species in the region to improve estimates of flora richness for the south-eastern mesoregion, the entire State of Mato Grosso and the phytogeographic domain of the Cerrado. The data we found indicate that poorly-collected municipalities, i.e. those with less than 200 plant samples recorded in herbarium collections, corresponded to 50% of the municipalities in the south-eastern mesoregion of Mato Grosso.

Poor knowledge of the vegetation of protected areas in the region still characterises herbaria collections in the region, making it difficult to assess the conservation status for endangered and endemic species. In fact, this difficulty is emphasised by the elevated proportion of species (~ 58%) with only one record observed for the south-eastern mesoregion of Mato Grosso. Studies with these taxa are needed to develop better conservation strategies.

The increase in floristc projects for this region, associated with the increase in qualified professionals in the area, is fundamental for conservation decision-making. In addition, the low collection rate, together with the high percentage of collections carried out by only a few collectors, demonstrates the urgency for increasing human resources to collect and identify plants.

## Supplementary Material

87CC97F8-4D75-576C-B943-1A9077571056 10.3897/BDJ.9.e72907.suppl1 Supplementary material 1 General species table Data type Occurrence data File: oo_591411.csv https://binary.pensoft.net/file/591411 Erica Campos, Massimo Bovini, Ariane Peixoto

## Figures and Tables

**Table 1.** Twenty main herbaria surveyed in this research with their respective acronyms according to Thiers (2020) (http://sweetgum.nybg.org/science/ih/).

HERBARIUM CODE	INSTITUTION
CEN	EMBRAPA Recursos Genéticos e Biotecnologia - EMBRAPA Cenargen
CGMS	Fundação Universidade Federal de Mato Grosso do Sul
CNMT	Universidade Federal de Mato Grosso – Campus Sinop
CPAP	EMBRAPA
ESA	Universidade de São Paulo – Campus Piracicaba
HPL	Jardim Botânico Plantarum
HUEFS	Universidade Estadual de Feira de Santana
ICN	Universidade Federal do Rio Grande do Sul
INPA	Instituto Nacional de Pesquisas da Amazônia
MBM	Museu Botânico Municipal
NYBG	New York Botanical Garden
PACA	Instituto Anchietano de Pesquisas/UNISINOS
RB	Jardim Botânico do Rio de Janeiro
SJRP	Universidade Estadual Paulista - Campus São José do Rio Preto
SP	Instituto de Botânica
SPF	Universidade de São Paulo
UB	Universidade de Brasília
UEC	Universidade Estadual de Campinas
UFG	Universidade Federal de Goiás
UFMT	Universidade Federal de Mato Grosso

**Table 2.** Collections in protected areas in the south-eastern mesoregion of Mato Grosso.

Protected areas	City	Area (km^2^)	Management	Number of specimens
R.P.P.N. Parque Ecológico João Basso	Rondonópolis	42.63	Private	81 (11 exclusive species)
A.P.A Parque Municipal Natural Nascentes do Rio Taquari	Alto Taquari	0.87	Municipal	01
Estrada Parque Cachoeira da Fumaça	Jaciara	6.98	State	01
Terra Indígena - Tadarimana	Rondonópolis	97.85	Federal	03 (02 species exclusive)
Overall	-	148.33	-	86

**Table 3.** Endangered species found in the south-eastern mesoregion of Mato Grosso. Threat Categories: Endangered (EN), Vulnerable (VU) and Near Threatened (NT) (CNC Flora 2020).

Species	Threat categories	Number of specimens
*Alstroemeriabrasiliensis* Spreng. (Alstroemeriaceae)	EN	1
*Anemopaegmaarvense* (Vell.) Stellfeld ex de Souza (Bignoniaceae)	EN	8
*Apuleialeiocarpa* (Vogel) J.F.Macbr. (Fabaceae)	VU	1
*Blepharodonhatschbachii* Fontella & Marquete (Apocynaceae)	EN	2
*Bowdichiavirgilioides* Kunth (Fabaceae)	NT	6
*Chamaecristadumalis* (Hoehne) H.S.Irwin & Barneby (Fabaceae)	NT	1
*Cyrtopodiumcaiapoense* L.C.Menezes (Fabaceae)	VU	1
*Diplopterysamplectens* (B.Gates) W.R.Anderson & C.C.Davis (Malpighiaceae)	NT	3
*Habenariaachalensis* Kraenzl. (Orchidaceae)	VU	1
*Handroanthusimpetiginosus* (Mart. ex DC.) Mattos (Bignoniaceae)	NT	1
*Hyptisfrondosa* S.Moore (Lamiaceae)	VU	3
*Hyptishamatidens* Epling & Játiva (Lamiaceae)	VU	1
*Micropholisgnaphaloclados* (Mart.) Pierre (Sapotaceae)	NT	1
*Pterandrahatschbachii* W.R.Anderson (Malpighiaceae)	VU	7
*Seemanniasylvatica* (Kunth) Hanst. (Gesneriaceae)	NT	1
*Sinningiadefoliata* (Malme) Chautems (Gesneriaceae)	VU	2
*Strophopappusbicolor* DC. (Asteraceae)	EN	1
*Strophopappusferrugineus* (Baker) R.Esteves (Asteraceae)	EN	1
*Talisiasubalbens* (Mart.) Radlk. (Sapindaceae)	VU	2
*Tibouchinapapyrus* (Pohl) Toledo (Melastomataceae)	NT	1

## References

[B7375963] APG (2016). An update of the Angiosperm Phylogeny Group classification for the orders and families of flowering plants: APG IV. Botanical Journal of the Linnean Society.

[B7375972] Barbosa MRV, Vieira A O Coleções de plantas vasculares: diagnóstico, desafios e estratégias de desenvolvimento.. http://www.cria.org.br/cgee/col/documentos.

[B7375980] Group BGF - The Brazil Flora (2015). Growing knowledge: an overview of seed plant diversity in Brazil. Rodriguésia.

[B7375989] Borges H B N, Silveira E A, Vendramin J N (2014). Flora arbórea de Mato Grosso: tipologias vegetais e suas espécies..

[B7375997] Campos E P, Silveira G L, Côrt A S Dalla, Nogueira L A S (2018). Florística e hábitos das espécies vegetais de um fragmento de cerrado em Rondonópolis, MT. Biodiversidade.

[B7376006] Brasil Flora do http://www.floradobrasil.jbrj.gov.br/.

[B7479171] Forzza R, Estevão L A, Dalcin Eduardo (2021). RB - Rio de Janeiro Botanical Garden Herbarium Collection. Version 84.226. Instituto de Pesquisas Jardim Botanico do Rio de Janeiro. Accessed via GBIF.org on 2021-09-24..

[B7376014] Gasper André Luís, Stehmann João Renato, Roque Nádia, Bigio Narcísio C., Sartori Ângela Lúcia Bagnatori, Grittz Guilherme Salgado (2020). Brazilian herbaria: an overview. Acta Botanica Brasilica.

[B7376041] Secretariat GBIF GBIF Backbone Taxonomy. Checklist dataset. https://www.gbif.org/dataset/d7dddbf4-2cf0-4f39-9b2a-bb099caae36c.

[B7476482] Greve M., Lykke A. M., Fagg C. W., Gereau R. E., Lewis G. P., Marchant R., Marshall A. R., Ndayishimiye J., Bogaert J., Svenning J. -C. (2016). Realising the potential of herbarium records for conservation biology. South African Journal of Botany.

[B7376057] MacDougall A. S, Loo J. A, Clayden S. R, Goltz J. G, Hinds H. R (1998). Defining conservation priorities for plant taxa in southeastern New Brunswick, Canada using herbarium records. Biological Conservation.

[B7376067] Mendonça R C, Felfili J M, Walter B M T, Jr M C Silva, Rezende A V, Filgueiras T S, Nogueira P E, Fagg C W, Sano S M, Almeida S P, Ribeiro J F (2008). Cerrado Ecologia e Flora.

[B7376092] Miranda L, Bastos H (2016). Atlas geográfico de Mato Grosso.

[B7376100] Monteiro S S, Siani A C (2009). A conservação de exsicatas em herbários: contribuição ao manejo e preservação. Revista Fitos.

[B7376131] Peixoto A L, Morim M P (2003). Coleções Botânicas: documentação da biodiversidade brasileira. Ciência e Cultura.

[B7376140] Peixoto A L, Barbosa M R V, Canhos D A l, Maia L C, Granato M, Rangel M (2009). Cultura Material e Patrimônio da Ciência e Tecnologia.

[B7376153] Pott A, Pott V J (1999). Flora do Pantanal, listagem atual de Fanerógamas. Anais II Simpósio sobre Recursos Naturais e Socioeconômicos do Pantanal.

[B7376167] Ratter J (1997). The brazilian cerrado vegetation and threats to its biodiversity. Annals of Botany.

[B7376176] Herbários Rede Brasileira de https://www.botanica.org.br/catalogo-da-rede-brasileira-de-herbarios/.

[B7376184] Virtual Reflora - Herbário http://reflora.jbrj.gov.br/reflora/herbarioVirtual/.

[B7376192] Sá Dominichi Miranda de, Sá Magali Romero, Lima Nísia Trindade (2008). Telégrafos e inventário do território no Brasil: as atividades científicas da Comissão Rondon (1907-1915). História, Ciências, Saúde-Manguinhos.

[B7482254] Ambiente SEMA - Secretaria de Estado de Meio Alertas de Desmatamento. http://sema.mt.gov.br/site/index.php/alertas-de-desmatamento.

[B7376201] Gestão SEPLAN - Secretaria de Estado e Planejamento e http://geoportal.seplan.mt.gov.br.

[B7376209] Shepherd G J (2003). Avaliação do estado do conhecimento da diversidade biológica do Brasil: plantas terrestres.

[B7479180] Silva Nilber Gonçalves, Martins Vera Lúcia Campos, Menezes Mariângela (2021). R - Herbário do Museu Nacional. Version 1.82. Museu Nacional / UFRJ. Accessed via GBIF.org on 2021-09-24..

[B7376385] Tarifa J R (2012). Clima do estado de Mato Grosso: análise e representação cartográfica.

[B7376393] Thiers B A global directory of public herbaria and associated staff. New York Botanical Garden’s Virtual Herbarium.. http://sweetgum.nybg.org./science/ih/.

[B7376401] Willis F, Moat J, Paton A (2003). Defining a role for herbarium data in Red List assesment: a case study of *Plectranthus* from eastern and southern tropical Africa.. Biodiversity and Conservation.

[B7376410] Windisch P G, Peralta D F, Araújo E L, Moura A N, Sampaio E V S B, Gestinari L M S, Carneiro J M T (2010). Biodiversidade, Conservação e Uso Sustentável da Flora do Brasil..

